# Knockdown of specific host factors protects against influenza virus-induced cell death

**DOI:** 10.1038/cddis.2013.296

**Published:** 2013-08-15

**Authors:** A T Tran, M N Rahim, C Ranadheera, A Kroeker, J P Cortens, K J Opanubi, J A Wilkins, K M Coombs

**Affiliations:** 1 Manitoba Centre for Proteomics and Systems Biology, Winnipeg, Manitoba R3E 3P4, Canada; 2 Department of Medical Microbiology and Infectious Diseases, University of Manitoba, Winnipeg, Manitoba R3E 0J6, Canada; 3 National Microbiology Laboratory, Public Health Agency of Canada, Winnipeg, Manitoba R3E 3R2, Canada; 4 Department of Physiology, University of Manitoba, Winnipeg, Manitoba R3E 3P4, Canada; 5 Manitoba Institute for Child Health, University of Manitoba, Winnipeg, Manitoba R3E 3P4, Canada; 6 Department of Internal Medicine, University of Manitoba, Winnipeg, Manitoba R3E 3P4, Canada; 7 Department of Biochemistry and Genetics, University of Manitoba, Winnipeg, Manitoba R3E 3P4, Canada; 8 Health Sciences Centre, Winnipeg, Manitoba, Canada

**Keywords:** RNAi arrays, host response, virus infection, apoptosis

## Abstract

Cell death is a characteristic consequence of cellular infection by influenza virus. Mounting evidence indicates the critical involvement of host-mediated cellular death pathways in promoting efficient influenza virus replication. Furthermore, it appears that many signaling pathways, such as NF-*κ*B, formerly suspected to solely promote cell survival, can also be manipulated to induce cell death. Current understanding of the cell death pathways involved in influenza virus-mediated cytopathology and in virus replication is limited. This study was designed to identify host genes that are required for influenza-induced cell death. The approach was to perform genome-wide lentiviral-mediated human gene silencing in A549 cells and determine which genes could be silenced to provide resistance to influenza-induced cell death. The assay proved to be highly reproducible with 138 genes being identified in independent screens. The results were independently validated using siRNA to each of these candidates. Graded protection was observed in this screen with the silencing of any of 19 genes, each providing >85% protection. Three gene products, TNFSF13 (APRIL), TNFSF12-TNFSF13 (TWE-PRIL) and USP47, were selected because of the high levels of protection conferred by their silencing. Protein and mRNA silencing and protection from influenza-induced cell death was confirmed using multiple shRNA clones and siRNA, indicating the specificity of the effects. USP47 knockdown prevented proper viral entry into the host cell, whereas TNFSF12-13/TNFSF13 knockdown blocked a late stage in viral replication. This screening approach offers the means to identify a large number of potential candidates for the analysis of viral-induced cell death. These results may also have much broader applicability in defining regulatory mechanisms involved in cell survival.

Viruses are obligate parasites that rely heavily on host cell machinery for productive replication. In addition to manipulating host factors that promote viral replication, influenza virus influences host signaling pathways that lead to extensive cell death and tissue damage during late stages of infection.^1^ This cell death may also contribute to the aberrant host immune responses that occur during disease progression.^1, 2, 3^ Although it was initially thought that virus-induced cytopathology resulted from active immune responses to viral infection, accumulating evidence strongly suggests that influenza virus manipulates the cell death signaling pathway(s) for their own benefit.^2, 3, 4, 5, 6, 7, 8, 9, 10^ Indeed, blockage of the cell death pathways leads to a significant decline in virus production.^6^

The present studies were undertaken to test the hypothesis that there are multiple human host cell genes that contribute to influenza-mediated cell death. We used a novel genome-wide lentivirus shRNAmir screen to identify genes required for influenza-induced cell death. In contrast to previously published influenza virus genome-wide screens,^11, 12, 13, 14, 15, 16, 17^ our approach used survival of infection with a cytotoxic dose of influenza as the selection condition (Table 1). Thus, the genes identified in our study were specifically required for virus-induced cell death.

A limited number of critically important genes required for influenza-induced cell death were selected for detailed analysis. The studies suggest that the assay provides a reliable means for selecting host genes involved in virus-mediated cytotoxicity. These genes may serve as potential targets for prevention of influenza virus-induced cell death.

## Results

### Identification of host factors required in virus-induced cell death

Our experimental approach was to perform a genome-wide silencing of host proteins using lentiviral-mediated transfer of shRNAmirs and to select for cells that survived influenza-induced cell death. The premise was that cell survivors had lost the expression of genes required for influenza-induced cell death. The identities of the genes that were silenced were subsequently determined by sequencing the shRNA that were present in the survivors.

**Figure 1:**
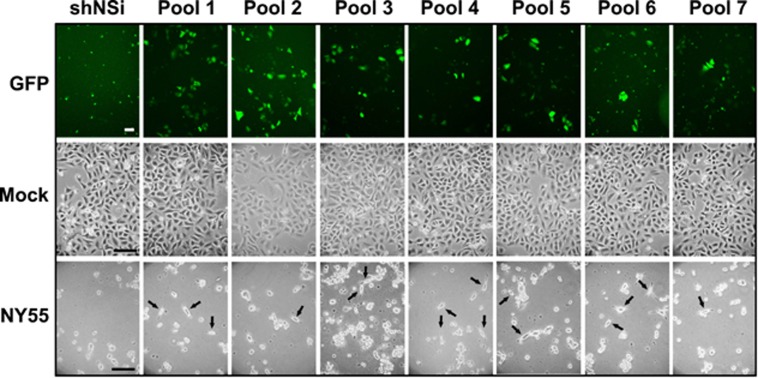
Global RNAi screen identifies genes required for virus-induced cell death. Images of transduced and infected cells were taken for each shRNA pool at 72 h after infection with influenza virus A/NY/55/2004(H3N2; NY55). Cytopathic effect of cells were examined with a Nikon Eclipse TE2000-S inverted microscrope and images obtained with a Canon PowerShot A700 digital camera. Shown are images representative of two independent replicates. Scale bar represents 100 *μ*M. GFP fluorescence was detected with Axio Observer.Z1 fitted with Plan-Apochromat 10 × /0.45 M27 objective (Carl Zeiss MicroImaging GmbH), AxioCamHR3 and AxioVision imaging software. Images were collected at 1388 × 1040-pixel resolution. The black arrows indicate surviving cells. The images were minimally rendered in Adobe Photoshop for publication purposes

Silencing was carried out with seven pools of lentivirus (∼10 000 clones/pool) in which each viral clone specifically targeted an mRNA. The transductions were done at a multiplicity of infection of 0.3 to reduce the risk of multiplying transducing cells with shRNAmir. The transduced cells were cultured for 48 h to allow silencing of target protein expression and subsequently infected with influenza virus strain A/New York/55/2004(H3N2; NY55) at a high multiplicity of infection (MOI) of 7 to ensure that more than 99% of the cells were infected. The infection was allowed to proceed for 72 h to allow for complete destruction of those cells that had either not been transduced or that had received a nonprotective shRNAmir (Figure 1). The surviving cells from each pool were harvested and the shRNAs were sequenced. Two independent screens were performed for each pool.

A small residual population of apparently resistant cells was consistently observed in each of the lentiviral-transduced populations after influenza infection. However, the vast majority of cells showed extensive virus-induced cytopathic effect (CPE) at 72 h post infection (h.p.i.) that manifested as rounded detached cells with subsequent death. These survivors were not observed in infected cells that had received nonsilencing control shRNAmir.

A total of 1256 potential gene targets were identified in at least one experiment. We chose to focus on the 138 highest confidence candidate genes that were identified in both screens. A detailed analysis of the 138 genes revealed that the sequences of 11 candidates could not be confidently assigned to a specific gene(s) in current genome models. This resulted in a list of 127 candidates for further analysis.

Only 2 of our 138 genes overlapped with genes identified in other influenza virus RNAi screens, *TRDMT1* and *GON4L*, which were identified by Konig *et al.*^18^ and Sui *et al.*,^19^ respectively (Supplementary Table S1). In addition, another 95 of ∼1100 potential gene targets that had been observed only once in our screens were identified in one or more of these previously published influenza siRNA screens (Supplementary Table S1). Our data suggest that each of more than 100 genes are required for influenza virus regulation of host cell death.

**Figure 2:**
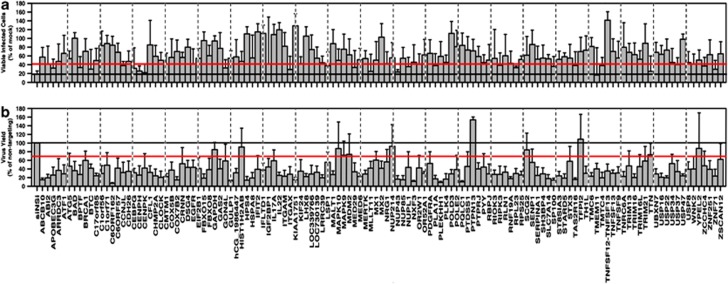
Secondary validation of candidate genes by siRNA array screen. (**a**) Viability of cells transfected with each of the indicated siRNAs was determined with WST-1 at 72 h after infection with NY55. (**b**) Virus replication was determined for cells transfected with each of the indicated siRNAs at 72 h.p.i. by titering infected supernatants on MDCK cells by plaque assay. Horizontal red line indicates statistical value of *P*<0.05. Error bars represent S.E.M. from three independent replicates

The target candidates were individually examined using siRNA to validate their protective effects. A549 cells were transfected with siRNA, cultured for 48 h and then infected with influenza virus. The cells were incubated for an additional 72 h and assessed for cell viability. There were graded levels of protection ranging from little or no protection to 100% protection relative to cells transduced with a nonsilencing control siRNA (Figure 2a). It was noteworthy that ∼2/3 of targets gave statistically significant protection (*P*≤0.05). These results indicated that the majority of genes that we identified in the shRNA screens were required for the induction of host cell death by influenza viruses.

There were 19 cases in which silencing of the gene resulted in almost complete protection (>85% survival) from virus-induced cell death (*P*<0.005; Figure 2a). Eight of these genes had the GO term apoptosis associated with them and two others were associated with the GO term DNA repair.

We also determined NY55 viral production at 72 h.p.i. (Figure 2b) in each of these knockdown (KD) cells. The majority of the genes identified were required for virus production. Knockdown of most genes resulted in significant reductions in virus titer. However, knockdown of 24 (19%) of the 127 genes identified in our high-throughput screens did not significantly reduce viral titer (Figure 2b) but these genes did show significant resistance to virus-induced cell death. This suggests that specific host genes are involved in the killing of the host cell during viral infection, and these can be independent of viral production.

### Two major host protein complexes play significant roles in influenza virus regulation of host cell death

**Figure 3:**
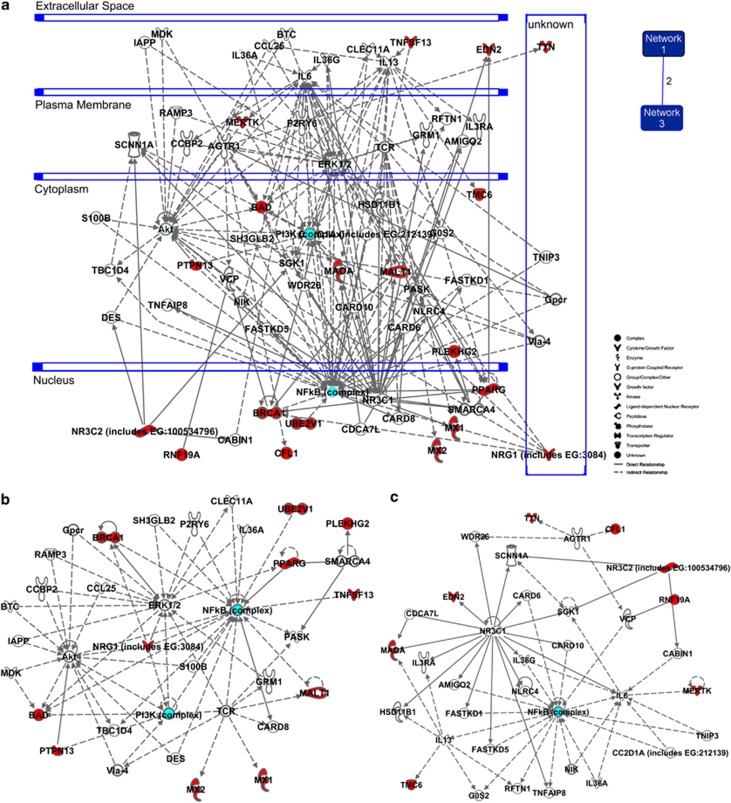
A total of 138 enriched genes from replicate screens were mapped to two major protein complexes. Interacting networks were determined with Ingenuity Pathway Analysis for the 70 annotated genes within the total list of 138 genes identified in the RNAi screens. Genes identified in our screens are indicated in red, unidentified genes are white and cyan highlights PI3K and NF-*κ*B complexes. (**a**) Both networks 1 and 3 (right inset) as a single merged network is shown. The number between the inset networks 1 and 3 schematic indicates overlapping genes. (**b** and **c**) Network 1 and Network 3, respectively, are shown

**Figure 4:**
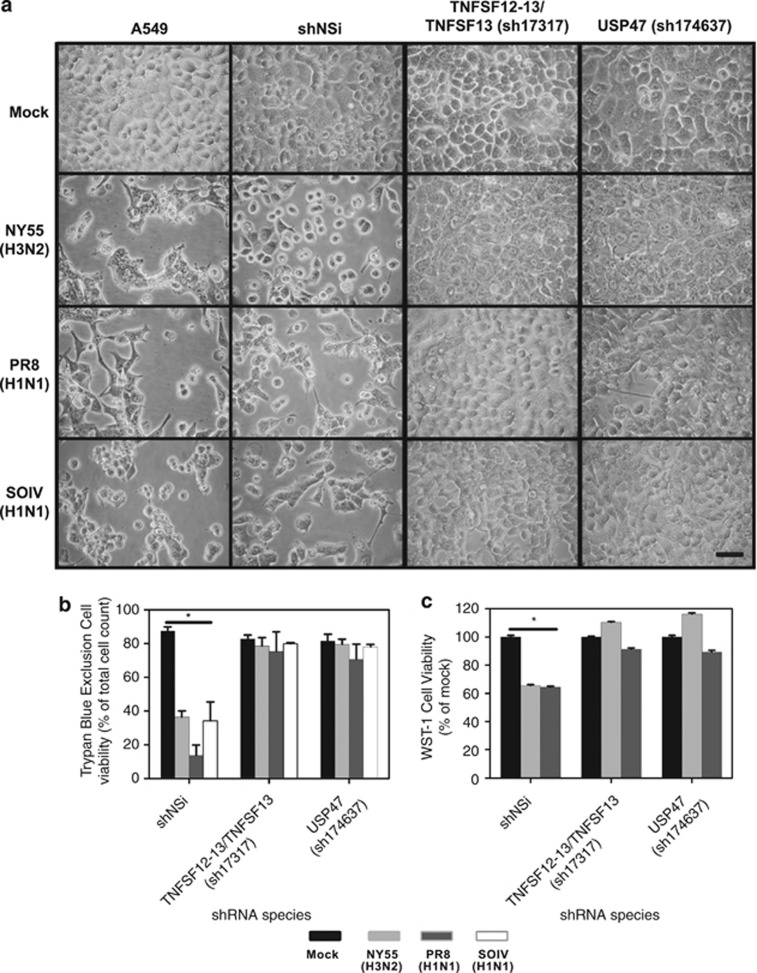
Reduced cytotoxicity and cell death by influenza virus infection in knockdown cells. Cytopathic effect in infected (**a**) TNFSF12-13/TNFSF13-shRNA or USP47-shRNA knockdown A549 cells that were infected with NY55, PR8 or SOIV. At 72 h.p.i., cytopathic effect of cells were examined with a Nikon Eclipse TE2000-S inverted microscrope and images obtained with a Canon PowerShot A700 digital camera. Shown are images representative of three independent replicates. Scale bar represents 100 *μ*M. Cell viability at 72 h after influenza virus infection was determined for shRNA-treated cells by (**b**) Trypan blue exclusion assay and by (**c**) WST-1 assay. A total of 200 cells were counted and the percentage of Trypan blue-excluding (viable) cells was determined. shNSi is nontargeting shRNA control. Shown is the mean from three independent replicates with error bars representing S.D. (*P<0.001)

We mapped the genes to two major protein complexes: PI3K and NF-*κ*B complexes (Figure 3a). Both complexes are induced during influenza virus replication.^20, 21^ More importantly, both PI3K and NF-*κ*B signaling cascades are involved in cell death and survival.^20, 22^ Other key host factors also involved in cell death and survival are Akt and ERK1/2 (Figure 3b). BAD (Bcl2 antagonist of cell death), an important pro-apoptotic protein identified in the screen, is involved in all four protein networks: PI3K, NF-*κ*B, Akt and ERK1/2 (Figure 4b). We recently showed that BAD is required for virus activation of the host apoptotic pathway.^10^

### TNFSF12-13, TNFSF13 and USP47 are required for influenza virus-mediated cell death

Three candidate genes, *USP47* (*ubiquitin-specific protease 47*), *TNFSF12-TNFSF13* (*tumor necrosis factor ligand superfamily member 12-13*) and *TNFSF13* (*tumor necrosis factor ligand superfamily member 13*), were selected for detailed analysis because of the high levels of protection from viral cytotoxicity that was conferred by silencing their gene products. These three genes also had been previously identified in an independent preliminary manual screen of two library pools, further suggesting the importance of these genes in influenza-induced cell death. TNFSF12-TNFSF13 was selected as it is a fusion protein generated from the *TNFSF12* and *TNFSF13* genes, raising the possibility that targeting either TNFSF13 or the fusion protein might affect cell survival.^23, 24^

Transient transfection with five different synthetic siRNAs individually targeting each of these genes resulted in >80% reduction of their mRNA levels relative to those treated with nonsilencing controls. In the case of USP47, protein levels were reduced to <10% of control levels (Supplementary Figure S1). Similarly, transduction with individual shRNA clones targeting each of these candidates also inhibited mRNA and protein expression. The analysis of TNFSF12-TNFSF13 and TNFSF13 expression was limited to mRNA expression because of the lack of suitable antibodies.

**Figure 5:**
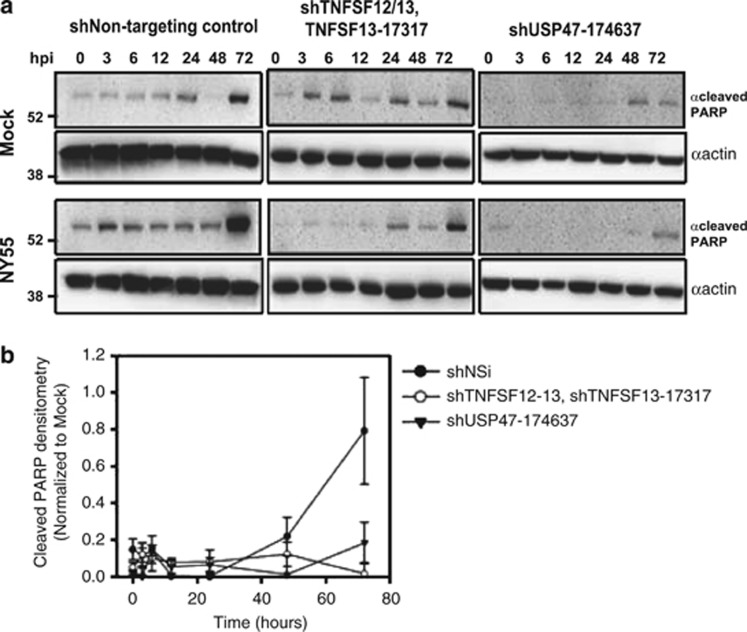
Cleavage of PARP is inhibited in influenza virus-infected knockdown cells. (**a**) Nontargeting shRNA control and various knockdown cells were infected with NY55 at MOI 3 and cells were harvested at specific time points. Whole-cell lysates were subjected to western blotting with antibodies to cleaved PARP. Mock is uninfected control. (**b**) Densitometry of cleaved PARP bands from western blots. *P<0.03. S.E.M. was determined from three replicate blots

It was of interest to assess the involvement of these proteins in the cytotoxic effects of other influenza strains because different influenza virus strains exhibit phenotypically dissimilar biologic activities. A549 cells were transduced with either nonsilencing control or specific shRNA targeting USP47 or TNFSF12/12-13. We used a single shRNA, sh17317, to target TNFSF13 and TNFSF12-13 as the supplier predicted that this would suppress both gene products. This construct inhibits the TNFSF13 region that is also required to generate TNFSF12-TNFSF13 that arises as a fusion protein derived from TNFSF12 and TNFSF13.^24^ Silencing of these proteins resulted in full protection of A549 cells from the cytotoxic effects of infection with influenza virus strains NY55, PR8 or SOIV (Figure 4). Moreover, we observed that activation of PARP, a factor involved in intrinsic apoptotic signaling, required active viral infection. When virus replication was inhibited in TNFSF12-13/TNFSF13 or USP47 KD cells, PARP cleavage was also significantly reduced (Figure 5). Densitometry showed PARP cleavage was reduced by more than 4- and 50-fold in USP47 and TNFSF12-13/TNFSF13 KD cells, respectively, compared with the shRNA nontargeting control at 72 h.p.i. (Figure 5b, *P*<0.03 shNSi). Faint bands indicative of PARP cleavage were observed in uninfected cells; a proportion of the population of cultured cells are normally known to undergo apoptosis.

### Knockdown of TNFSF12-13, TNFSF13 and USP47 proteins inhibit different stages of influenza virus replication

**Figure 6:**
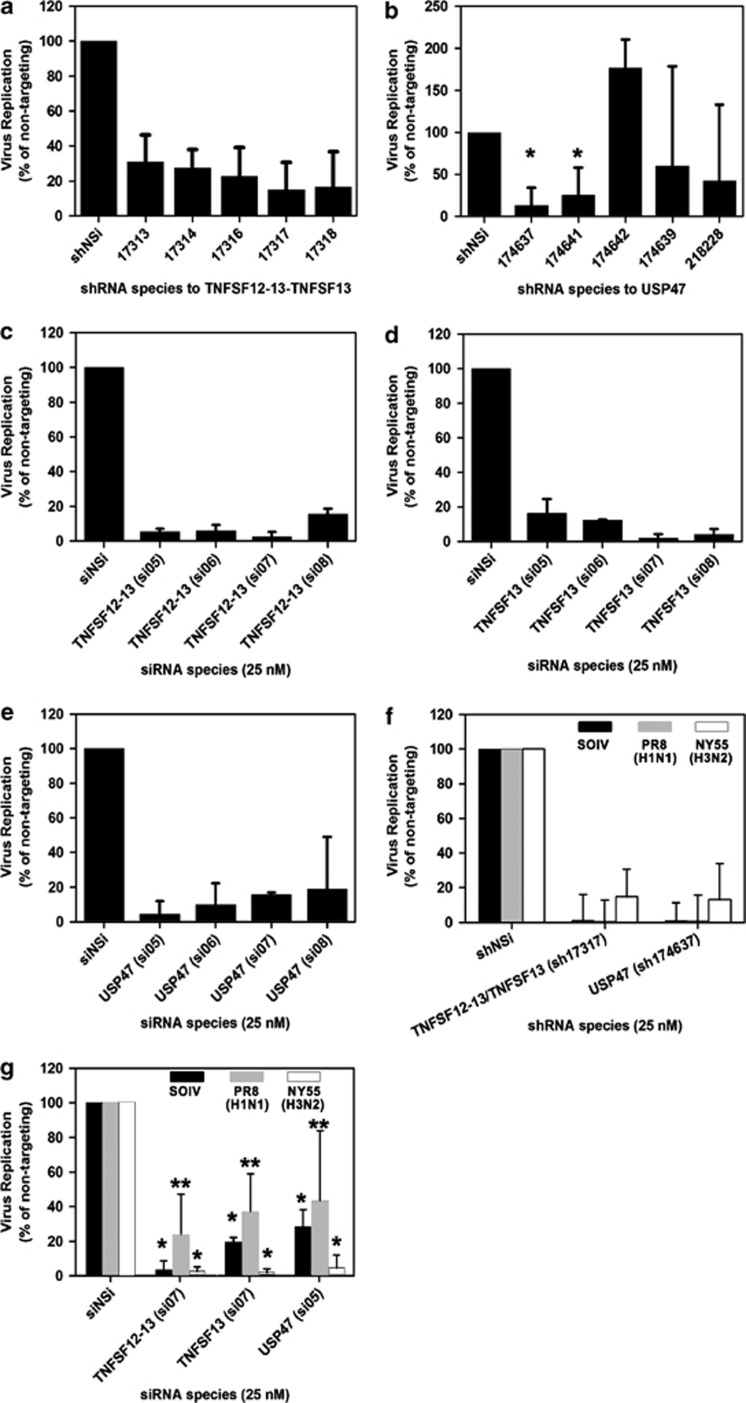
Inhibition of influenza virus replication in knockdown cells. Virus replication was determined at 72 h.p.i. for NY55 in (**a**) TNFSF12-13/TNFSF13 (*P*<0.001) or (**b**) USP47 (*P<0.05) shRNA KD cells. NY55 replication was determined in siRNA KD cells for (**c**) TNFSF12-13, (**d**) TNFSF13 or (**e**) USP47 (*P*<0.001). Virus replication in PR8- and SOIV-infected TNFSF12-13, TNFSF13 or USP47 (**f**) shRNA KD (*P*<0.001) and (**g**) siRNA KD cells (*P<0.001, **P<0.05). NSi is nontargeting shRNA or siRNA control. Error bars represent the mean+S.E.M. from three independent replicates

We previously showed (Figure 2) that some genes identified in our screen support virus-induced cell death but not all identified genes necessarily influence the ability of the virus to produce progeny virions. As one of our ultimate goals is to identify genes involved in both cell killing and virion production, we determined whether TNFSF12-13, TNFSF13 and USP47 KD would affect influenza virus replication. NY55 virus production was reduced to <30% of the nontargeting control for all five shRNA species that target TNFSF12-13/TNSF13 (Figure 6a, *P*<0.001), and two of the five shRNA species targeting the USP47 transcript showed significant reduction in viral titer to <32% of the nontargeting control (Figure 6b, *P*=0.006). We are uncertain why shRNA-174642 appeared to result in increased viral production; the possible explanations include unexpected consequences resulting from the site of shRNA integration into the host genome, or this specific shRNA sequence had a high off-target effect. For these reasons, we also repeated the experiments using transient transfection with siRNA duplexes that individually target TNFSF12-13, TNFSF13 or USP47. Viral production in siRNA-transfected cells showed reduced virus titer, which corroborates that observed with shRNA-stable KD cells. siRNA KD of TNFSF12-13 resulted in reduced viral replication to <15% of the nontargeting siRNA control for all four siRNA species tested (Figure 6c, *P*<0.001). Similarly, virus production was inhibited in TNFSF13 siRNA KD cells to <16% of the nontargeting control (Figure 6d, *P*<0.001) and viral replication was <19% of the control in USP47 siRNA KD cells (Figure 6e, *P*<0.001).

shRNA KD of TNFSF12-13, TNFSF13 and USP47 also inhibited viral replication of other viral subtypes, namely PR8 and SOIV, to <10% of the nontargeting control (Figure 6f, *P*<0.001). Similarly, we observed significant reduction of both SOIV and PR8 viral production in all three siRNA KD cell lines; <30% of control for SOIV (*P*<0.001) and <45% of the control for PR8 (*P*<0.05) (Figure 6g). We confirmed that the reduction in virus titers were not due to RNAi effects on cell viability (Supplementary Figure S2).

### Knockdown of USP47 attenuates virus entry and TNFSF12-13 and TNFSF13 knockdown attenuate late stages of viral replication

**Figure 7:**
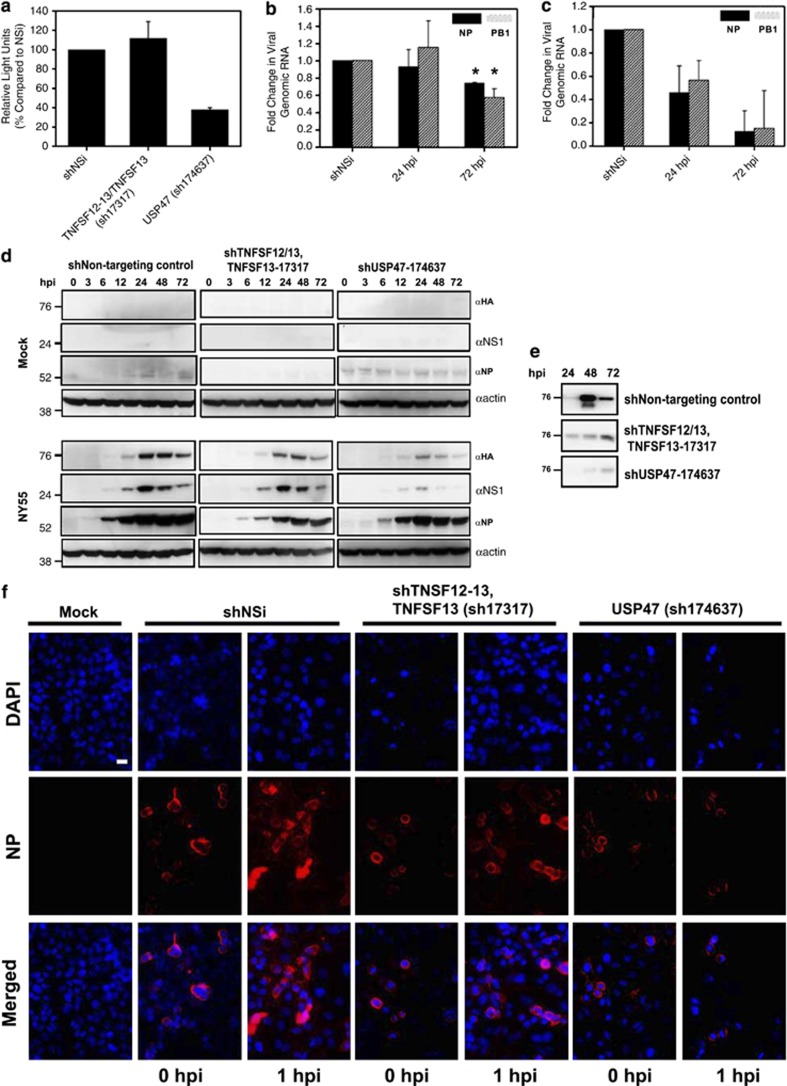
USP47 and TFNSF12-13/TNFSF13 knockdowns inhibit different stages of viral replication. (**a**) Luciferase mini-genome assay to assess viral transcription in indicated cells. Real-time PCR was used to determine viral genome replication for infected (**b**) TNFSF12-13 (*P<0.001) and (**c**) USP47 shRNA knockdown cells (*P*<0.05). (**d**) Cells were infected with NY55 at MOI 3 and whole-cell lysate was obtained at specified time points. Western blot was probed with *α*-NS1, *α*-NP and *α*-HA antibodies. (**e**) Cells were infected with NY55 and supernatants were collected at the specified time points. Viruses were pelleted from the supernatant, lysed and blotted with *α*-HA antibody. (f) Various indicated cells were pretreated with 1 mM cycloheximide and then infected with NY55 at an MOI of 100. Cells were fixed at the indicated times, stained for NP protein and images obtained

**Figure 8:**
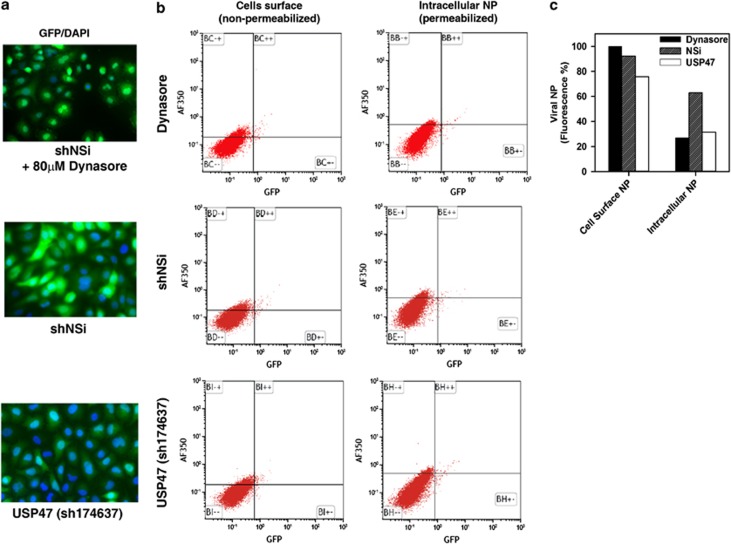
USP47 knockdown inhibits influenza virus entry. (**a**) Fluorescence detection of GFP insert in cells under various experimental conditions. (**b**) Virus entry was determined by flow cytometry with permeabilized (intracellular) and nonpermeabilized (cell surface) USP47 knockdown cells infected with NY55 at an MOI of 50. Virus entry was determined by detection with anti-NP mouse monoclonal antibody conjugated with Alexa Fluor 350. Dynasore, a known inhibitor of influenza virus entry, was used as a positive control. shNSi indicates shRNA nontargeting control. (**c**) Quantitation of the two upper quandrants (+/− and ++) after each condition. Data are representative of three replicates

Viral transcription, genomic replication and protein translation were initially assayed to determine whether viral replication stages that may be affected in TNFSF12-13/TNFSF13 and USP47 KD cells were relatively early or late. Luciferase mini-genome assays were carried out to assess viral transcription, which involves transfecting A549 cells with a luciferase-expressing construct. Luciferase expression is directly proportional to viral transcription. Viral genomic replication was determined with real-time PCR of viral genes NP and PB1. Western blot was used to determine levels of viral protein production in the knockdown cells. Results from these assays showed significant reduction of viral transcription (Figure 7a, *P*<0.001), viral genomic replication (Figures 7b and c, *P*<0.05) and viral protein production (Figure 7d) in USP47 KD cells but not in TNFSF12-13/TNFSF13 KD cells. Thus, the defect that causes reduced viral titers in TNFSF12-13/TNFSF13 KD cells appears to occur after this step in virus replication, whereas the defect that causes reduced viral titers in USP47 KD cells occurs before genomic replication and viral protein translation. To determine whether viral entry may be blocked in USP47 KD cells, we assessed viral entry by immunofluorescence. Cells were pretreated with cycloheximide to block viral protein production, thereby allowing us to detect infecting viral particles only. Our data show that after 1 h.p.i., virus particles remained attached to the periphery of the host cell in USP47 KD cells (Figure 7f). However, in shRNA nontargeting control and TNFSF12-13/TNFSF13 KD cells, a portion of incoming virus NP protein (part of the viral ribonucleoprotein complex) has entered the nucleus. This suggests that USP47 activity is required for viral entry, which is blocked in USP47-deficient cells. This suspected defect was confirmed and quantified by analyzing permeabilized and nonpermeabilized cells by flow cytometry after various treatments. We used dynasore, a dynamin inhibitor known to block influenza virus entry in the absence of serum,^25^ as a positive control for influenza virus entry inhibition. Our knockdown cells all express a low level of GFP from the shRNA construct inserted into the host genome (Figure 8a). This fluorescence was not efficiently detected by flow cytometry (Figure 8b), although analysis of the data indicated that viral entry in USP47-shRNA KD cells was reduced by more than twofold compared with the nontargeting control (Figure 8c). Collectively, the immunofluorescence and flow cytometry data indicate that USP47 knockdown affects influenza virus entry.

We also considered virus particle release into the supernatant to pinpoint the stage of viral inhibition in TNFSF12-13/TNFSF13 KD cells. We observed significant reduction in virus particles released into the supernatant in infected TNFSF12-13/TNFSF13 KD cells, as measured by HA quantity (Figure 7e), which suggest that either viral release into the supernatant or viral assembly is affected in these cells. Reduced viral particle production was also observed in USP47 KD cells, which was to be expected as virus replication is blocked at an early stage in USP47-deficient cells. Overall, our results indicate that these host factors play significant roles in promoting different aspects of influenza virus replication and virus-induced cell death for different virus subtypes.

## Discussion

Our screen reproducibly identified a set of human genes that are required for influenza-induced cell death. The requirement for these candidate genes was independently confirmed using synthetic siRNA-mediated silencing. Detailed analysis of three proteins, USP47, TNFSF12 and TNFSF12/TNFSF12-13, confirmed that knocking down these proteins resulted in >85% protection from influenza-induced cell death.

The current study was based on a functional screen that examined the impact of protein expression knockdown on influenza virus-induced cell death. There have been several genome-wide silencing studies using reporter systems as surrogates of viral infection.^11, 12, 13, 14, 15, 16^ However, there has been relatively little overlap between any of the various previous studies.^12^ Similarly, there is a very low level of overlap between the genes identified in our study and those reported by others. However, it is noteworthy that a detailed comparison of our results with these other studies indicates the highest degree of concordance between our study and that of Shapira *et al.*^11^ There were 38 commonly identified targets (Supplementary Table S1) and 2 of the commonly identified genes (*POLD3* and *TRIM21*) were among those that confer >85% cell survival in our assay. Many of the candidates identified by others may indeed be important for regulation of viral protein production, but given that our study is based on cell survival, it may be that these could go undetected if they did not alter survival to infection such as that required for our screen. An obvious limitation to any genome-wide approach is the inability to examine the roles of any gene(s) that are required for cell survival in general, as silencing of these would eliminate the cells from the population.

We chose to initially focus on those genes that were reproducibly identified in both screens as these represented the most confident gene candidates. The siRNA transfection experiments were used as a confirmatory orthogonal method of gene knockdown. It was confirmed that knockdown of 121 of the 127 targets tested protected cells from influenza infection-induced cell death. However, it was noted that there was considerable variation in the levels of protection achieved with this approach. This may be a reflection of differences in the levels of silencing achieved by the siRNA and the shRNA directed against the same mRNAs because of targeting or duration of inhibitors. Alternatively, it could be that some of the targets identified in the original screen were detected because they reduced, rather than inhibited, the rate of cell killing by the virus. Despite the fact that loss of many of these candidates was fully protective, it is clear that the presence of these proteins affects the survival of influenza-infected cells. These observations demonstrated that the effects of knockdown were not because of off-target effects related to the inhibitory RNA or the method of their introduction into the cell (i.e., transduction *versus* transfection).

A group of 19 proteins was identified whose knockdown resulted in >85% protection from virus-induced death. These proteins did not display any significant molecular interaction as assessed by STRING analysis. It was noteworthy that the majority of these 19 proteins were GO annotated as being involved in aspects of regulation of apoptosis or cell proliferation/differentiation. Two of the genes, *USP47* and *TNFSF12-13*, in this highly protective group were selected for more detailed analysis. Another protein, TNFSF13, was also examined because silencing of this protein was found to give significant antiviral protection and this gene contributes to the generation of a fusion protein arising from splicing of the TNFSF12 and TNFSF13 mRNAs.^24^ Knockdown of each target by multiple siRNA or shRNAmir was confirmed at the message or protein levels. The independent expression of the knockdown of each of these proteins resulted in complete protection from infection-induced cell death. These effects did not appear to be virus strain specific as similar observations were made with three different influenza subtypes. Based on these results, it would appear that there are commonalities in the cytotoxicity induced by several influenza virus strains.

USP47 is a 157-kDa ubiquitin carboxyl-terminal hydrolase that deubiquinates monoubiquitinated DNA polymerase-*β*.^26^ It was recently reported that USP47 interacts with the E3 ubiquitin ligase, Skp1/Cul1/F-box protein *β*-transducin repeat-containing protein (SCF*β*^-Trcp^),^27^ and regulates the steady-state levels of DNA polymerase-*β*.^26^ Interestingly, both of these studies reported that silencing of USP47 in HEK293T cells^27^ and HeLa cells^26^ led to a decrease in cell survival and proliferation. Significantly, these effects were observed under conditions that induced DNA damage. However, in our studies, stable knockdown of USP47 in A549 did not influence cell survival. These observations are in agreement with others who reported that knockdown of this protein in A549 cells had no effect on cell viability and proliferation.^28^ Although these observations could suggest that USP47 functions are cell-type dependent, the possibility remains that USP47 is required for cellular protection under physiological stress conditions.

TNFSF13 (APRIL, TALL-2, TRDL-1 or CD256) and TNFSF12-TNFSF13 (TWE-PRIL) are members of the tumor necrosis factor ligand superfamily. TNFSF13, a soluble protein, is overexpressed in a number of hematological and solid tumors and its increased expression is associated with poor prognosis.^29^ TNFSF12-TNFSF13 derives from splicing of the mRNAs of TNFSF12 and TNFSF13 to generate a fusion protein. TNFSF12-TNFSF13 is an integral membrane protein with the intracellular, transmembrane and a short extracellular region derived from TNFSF12. The remaining extracellular region is derived from the TNFSF13 receptor-binding region. TNFSF12-TNFSF13 appears to display similar biological properties to TNFSF13, although the relationship between these proteins has not been extensively characterized. As our screens only identified loss of TNFSF13 or TNFSF12-TNFSF13 resulting in protection from influenza-induced cell death, this could suggest that TNFSF12 alone was not responsible for the cytotoxicity associated with influenza infection.

We have determined that knockdown of USP47 prevented proper entry of the virus into the host cell, which resulted in early attenuation of influenza virus infection. This suggests that USP47 activity is required for viral entry. Influenza virus entry into host cells has been associated with the ubiquitin signaling pathway.^30^ Here we have identified a specific host factor that is involved. On the other hand, knockdown of TNFSF12-13/TNFSF13 did not block viral transcription, translation and viral genomic replication, but viral egress was significantly reduced. This suggests that TNFSF12-13/TNFSF13 activity is required at a late stage of viral replication such as virus assembly or virus release.

We, and others, have previously reported that influenza virus induces apoptosis upon infection.^10, 31, 32, 33^ Moreover, influenza virus induces cell death through the apoptotic pathway and not by necrosis.^31, 32, 33^ In further support of this, we reported that viral infection resulted in the activation of several factors of the intrinsic apoptotic pathway,^10^ including PARP, a substrate of caspase-3. Our data showed that PARP cleavage was significantly reduced in TNFSF12-13/TNFSF13 and USP47 KD cells, which suggests that apoptosis was not activated in these knockdown cells. In addition, we have shown that silencing the expression of BAD, another of the candidates identified in this screen, protected host cells from influenza virus-induced death.^34^ It was also noted that virus production was markedly reduced in cells with reduced expression of BAD. These results suggest that the activation of apoptotic pathways can be regulated during influenza infection and point to the need for apoptosis during normal viral production. This interpretation was consistent with the observation that 8 of the 19 genes that appeared to be most essential for influenza-induced cell death were annotated in gene ontology as being involved in apoptosis or regulation.

In summary, we have described a robust genome-wide screen for identification of genes required for influenza-mediated cytotoxicity. The results were independently confirmed using alternate silencing approaches. The screen has identified a large number of potential candidates for the analysis of viral-induced cell death. These results may have much broader applicability in defining regulatory mechanisms involved in cell survival.

## Materials and Methods

### Human whole-genome screen

A549 cells were transduced at an MOI of 0.3 PFU per cell with each of 7 Decode RNA GIPZ Lentiviral Positive Screening Library pools according to the manufacturer's protocol (Thermo Scientific Open Biosystems, Ottawa, ON, Canada). After 48 h, cells were washed twice with 1 × phosphate-buffered saline (PBS) and infected with NY55 at an MOI of 7 PFU. At 72 h.p.i., cells were washed twice with 1 × PBS and harvested. Genomic DNA was isolated by phenol/chloroform extraction followed by ethanol precipitation.

### PCR and real-time PCR

PCR was carried out on isolated genomic DNA using Expand High Fidelity polymerase mix (Roche, Laval, QC, Canada) and the product was purified from polyacrylamide gels. PCR primers used for Illumina sequencing are proprietary properties of Illumina, Inc. (San Diego, CA, USA) and Canada's Michael Smith Genome Sciences Centre (MSGSC; Vancouver, British Columbia, Canada). Pooled cDNA was sequenced by high-throughput Illumina sequencing technology at MSGSC. Target cellular mRNA knockdown efficiency and viral genomic replication was determined by quantitative real-time PCR (qRT-PCR). Total mRNA was isolated using RNeasy Mini Kit (QIAGEN, Toronto, ON, Canada) according to the manufacturer's protocol. Purified mRNA at 500 ng was used to generate cDNA with random hexamer primers (Applied Biosystems, Life Technologies, Burlington, ON, Canada) and SuperScript II Reverse Transcriptase (Invitrogen, Life Technologies) according to the manufacturer's protocol. The qRT-PCR reaction mix (25 *μ*l) consists of: 12.5 *μ*l of SYBR Green PCR Master Mix (Invitrogen), 0.5 *μ*l cDNA template and 1 *μ*l of each of 100 *μ*M forward and reverse primers (Supplementary Table S2). Reactions were run on Applied Biosystems 7300 Real-Time PCR System. The cycling conditions were as follows: 50^o^C for 2 min, 95^o^C for 2 min and 50 cycles of 95^o^C for 15 s and 60^o^C for 30 s. Ct values were normalized to 18S rRNA control and compared with nontargeting (‘nonsilencing') sh/siRNA negative control.

### Lentivirus packaging and transduction

Individual human shRNAmir lentiviral clones (Thermo Scientific Open Biosystems) were prepared and isolated as previously described.^34^ Briefly, individual shRNAs (Supplementary Table S2) were packaged into lentivirus particles by cotransfection of each shRNAmir with pMD2.G and psPAX2 (Addgene (Cambridge, MA, USA) plasmids 12259 and 12260, respectively) in individual sets of HEK-293T cells. Stable KD A549 cells were produced by transducing with lentivirus at an MOI of 0.5. At 72 h post transduction, 3 *μ*g/ml puromycin (Sigma, Oakville, ON, Canada) was added to the media. Cells were passaged over a 2-week period in puromycin-supplemented completed 1 × DMEM media to select transductants before they were infected with virus.

### siRNA transfection

siRNA transfections were carried out as previously described.^34^ Sets of A549 cells were treated with 25 nM of each of the four ON-Targetplus siRNAs (Dharmacon, Thermo Scientific, Ottawa, ON, Canada) targeting each of the *TNFSF12-13*, *TNFSF13* and *USP47* genes (Supplementary Tables S3 and S4). siRNAs were introduced into cells with Lipofectamine RNAiMAX (Life Technologies). Each cell set was retreated with the same siRNA 24 h later. After a further 24 h, cells were infected with virus.

### Influenza virus infection and plaque assay

Sets of transduced or transfected A549 cells were infected with influenza virus strains A/New York/55/2004(H3N2; NY55) at an MOI of 1 (shRNA) or 0.1 (siRNA) PFU/cell, or with A/PR/8/34(H1N1; PR8) or with A/California/7/09 (H1N1; SOIV) at an MOI of 0.1. At 72 h.p.i., supernatants were harvested and virus yield was titrated by plaque assay on MDCK cells. For shRNA genomic screen, transduced cells were infected with NY55 at an MOI of 7 for 72 h. All influenza virus infections occurred at 35^o^C in 5% CO_2_ humidified environment, including the plaque assay.

### Cell viability

Cell viability was determined using Cell Proliferation Reagent WST-1 (Roche) according to the manufacturer's protocol or Trypan blue exclusion assay. For Trypan blue exclusion assay, ∼1 × 10^6^ infected or uninfected cells were stained with 20 *μ*l of Trypan blue solution and ∼14 *μ*l of the stained cells were placed on a hemocytometer. A total of 200 cells were counted and the percentage of viable cells was calculated with the following formula:


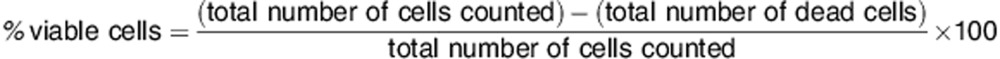


### Virus entry and immunofluorescence

Cells were pretreated with 1 mM protein synthesis inhibitor cycloheximide and then prechilled at 4^o^C before virus adsorption. After virus adsorption, cells were incubated at 35^o^C. Cells were fixed with 4% paraformaldehyde at 0 and 1 h.p.i., permeabilized with Triton X-100 and probed with mouse anti-NP mAb. Immunofluorescence microscopy was performed with Axio Observer.Z1 fitted with EC Plan-Neofluar 40 × /0.75 M27 objective (Carl Zeiss MicroImaging GmbH, Göttingen, Germany), AxioCamHR3 and AxioVision imaging software (Carl Zeiss MicroImaging GmbH). Images were collected at 1388 × 1040-pixel resolution. The images were rendered in Adobe Photoshop (Adobe Systems Canada, Ottawa, ON, Canada).

### Flow cytometry

Cells were pre-chilled on ice before and during virus adsorption. After virus adsorption, cells were incubated at 35^o^C for 5 h. For intracellular antigen detection, cells were fixed with 70% ethanol (v/v), permeabilized with 0.25% Triton-X-100 and probed with mouse anti-NP mAb conjugated with Alexa Fluor 350 (Invitrogen). For surface antigen detection, cells were blocked with 1 × PBS/1% BSA (w/v), probed with mouse anti-NP mAb conjugated with Alexa Fluor 350 (Invitrogen), and lastly fixed with 70% ethanol (v/v). Samples were analyzed with a Beckman Coulter MoFlo XDP Cell Sorter using Kaluza Analysis software (Beckman Coulter Canada, LP., Mississauga, ON, Canada). Dynasore (Sigma Aldrich, Oakville, ON, Canada) dynamin inhibitor was used as a positive control. Cells were pretreated with 80 *μ*M Dynasore in the absence of serum for 1 h, and the inhibitor was included for all stages of infection thereafter.

### Luciferase mini-genome assays

A549 cells were transfected with PR8-based PA, PB1, PB2 and NP expressing plasmids (pCAGGS), WSN-luciferase mini-genome and pRenilla, essentially as previously described.^35^ Luciferase activity was determined 48 h after transfection.

### Western blot

Whole-cell lysates were obtained by lysing cells in RIPA buffer (50 mM Tris, pH 7.4; 150 mM NaCl; 1 mM EDTA; 1% Triton X-100; 0.1% SDS) with complete protease inhibitor (Roche). Then, 20 *μ*g of each lysate was separated by SDS-PAGE, transferred to Immobilon-P PVDF membranes (Millipore, Billerica, MA, USA) and blotted with mouse monoclonal *α*-NS1 (3F5), mouse monoclonal *α*-NP (F26-9;^36^ a gift from Dr. Mingyi Li, National Microbiology Laboratory, Winnipeg, MB, Canada), rabbit polyclonal *α*-HA (Rockland, Gilbertsville, PA, USA) and rabbit polyclonal *α*-cleaved PARP (Asp214, New England Biolabs, Ltd., Whitby, ON, Canada). Blot images were obtained with Alpha Innotech FluorChem Q Imaging System (Protein Simple, Santa Clara, CA, USA) and processed using Adobe Photoshop. Densitometry was determined using an Alpha Innotech FluorChem Q Imaging System.

### Statistics

Statistical significance was calculated using Student's *t*-test.

## Figures and Tables

**Table 1:** RNAi screen parameters from different published influenza RNAi screens

	**Tran** ***et al*****. (this study)**	**Shapira*****et al***.^11^	**Konig*****et al***.^14^	**Karlas*****et al***.^13^	**Brass*****et al***.^17^	**Sui*****et al***.^15^	**Hao*****et al***.^16^
Approach	RNAi screen	Yeast-2-hybrid• RNAi screen	RNAi screen	RNAi screen	RNAi screen	Random homozygous gene perturbation	RNAi screen
Screen parameter	Selection of surviving cells after influenza infection	Level of *Renilla* luciferase expression in 293T reporter cells	Level of *Renilla* luciferase expression	Level of *Renilla* luciferase expression in 293T reporter cells	Stained for surface expression of HA	Host gene perturbation	Level of reporter gene expression
End point (h.p.i.)	72 h.p.i.	48 h.p.i.	36 h.p.i.	24 h.p.i.	12 h.p.i.	48 h.p.i.	24–48 h.p.i.
No. of genes screened	21,415	1745	19 628	22 843	17 877	NA	13 071
Delivery method	Lentiviral	Transfection	Transfection	Transfection	Transfection	Lentiviral	Infection with recombinant virus
Total candidates	1256 Genes	616 genes	295 Genes	287 Genes	312 Genes	110 Clones	110
Genes in biological replicates	138	NA	NA	NA	NA	NA	NA
Virus used	NY55 (H3N2), PR8 (H3N2), pandemic California (H1N1)	PR8 (H1N1), Udorn (H3N2)	Recombinant WSN (H1N1), HA-luciferase	WSN (H1N1)	PR8 (H1N1)	Udorn (H3N2)	Recombinant WSN (H1N1) with VSV G protein and NA-luciferase
Cell line used in screen	A549 cells	293T and HBEC cells	A549 cells	A549 and 293T cells	U2OS cells	MDCK cells	Drosophila cells

NA, not available

## Abbreviations

BAD: Bcl2 antagonist of cell death
CPE: cytopathic effect
h.p.i.: hours post infection
PBS: phosphate-buffered saline
qRT-PCR: quantitative real-time PCR
SCF^*β*-Trcp^: Skp1/Cul1/F-box protein *β*-transducin repeat-containing protein
TNFSF13: tumor necrosis factor ligand superfamily member 13
TNFSF12-13: tumor necrosis factor ligand superfamily member 12-13
USP47: ubiquitin-specific protease 47
