# Supplementary figures and images for: Knockdown of specific host factors protects against influenza virus-induced cell death
Source: Cell Death Dis. 2013 Aug 15;4(8):e769–. doi: 10.1038/cddis.2013.296 (PMC3763457; doi:10.1038/cddis.2013.296)

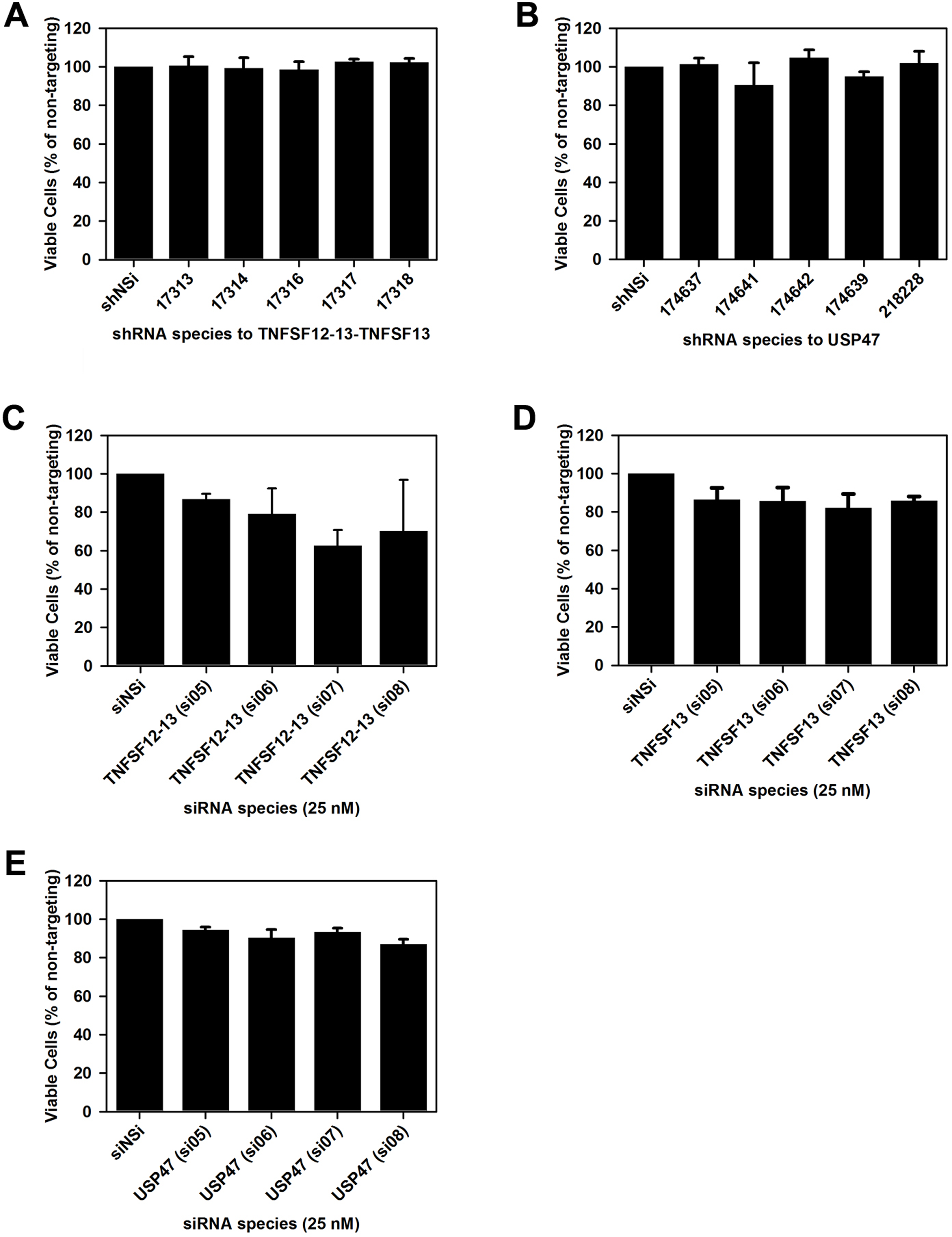

Supplement: Supplementary Figure S1 [file cddis2013296x1.tif]

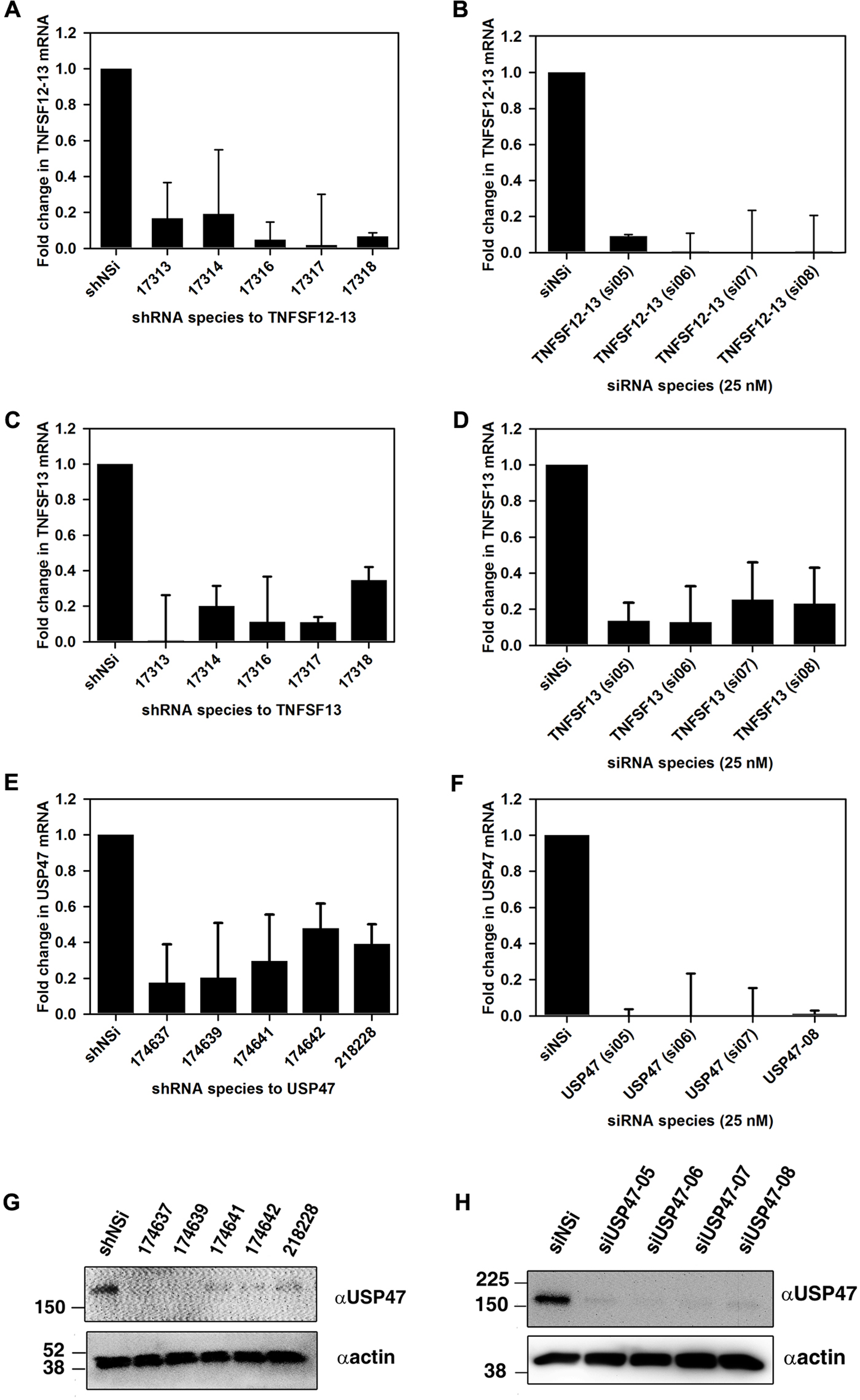

Supplement: Supplementary Figure S2 [file cddis2013296x2.tif]
