# Supplementary material for: Knockdown of specific host factors protects against influenza virus-induced cell death
Source: Cell Death Dis. 2013 Aug 15;4(8):e769–. doi: 10.1038/cddis.2013.296 (PMC3763457; doi:10.1038/cddis.2013.296)
Supplement: Supplementary Table S1 [file cddis2013296x3.pdf]

Table S1. Overlap between various Influenza virus RNAi screens

	Tran (this study)	Shapira[11]	Konig[14]	Karlas[13]	Brass[24]	Sui[15]	Hao[16]
Tran		38	20	18	9	6	6
Shapira			16	8	16	5	5
Konig				33	10	2	7
Karlas					7	2	6
Brass						2	11
Sui							0
Hao							

Specific genes identified in overlapping studies

ABCC10	ACTC1	ABCB10	ATF1	BPTF	DPF2
AMHR2	AKAP13	CD58	CRNKL1	C21orf33	HSPA8
APOBEC3G	BMPR1B	CNGB1	DCLRE1A	FBXL17	NUP43
ARMCX5	C5orf38	DERL3	GSK3A	GON4L	NXT2
C21orf33	CCL13	FHOD1	NUPL1	PKD2L2	PSMB4
CALM1 (includes others)	CDC42BPA	IGSF1	PAGE5	SLC30A9	RPS3A
CDKN2AIP	CLOCK	IL1A	PPARA		
CLEC2B	DCLK1	LOC100510692/NAIP	STARD5		
DHCR24	EPHA7	MDM2	ZNF154		
FBXO34FGFR2	MED6				
FGFR2IGSF1	MSRA				
HIST1H2AC	KHSRP	NXF3			
HMGA2MDM2	P2RY12				
IFIT2NUP214	RBM3				
IL1RAPPDK3	SERPINA1				
IRF6 PLK4	SIGMAR1				
ITPKBSCN8A	TK2				
LYN SGK196	TRIM21				
MAPK13	SIGMAR1				
NOD1TRDMT1					
NR4A2					
PDGFRA					
PHF3					
PNMA1					
POLD3					
PPP2R2B					
PQLC1					
PTPN6					
RIOK3					
RNF44					
RP2					
SAMHD1					
SRSF6					
SRSF7					
STARD5					
TOPORS					
TRIM21					
WTAP					
