# Supplementary material for: Knockdown of specific host factors protects against influenza virus-induced cell death
Source: Cell Death Dis. 2013 Aug 15;4(8):e769–. doi: 10.1038/cddis.2013.296 (PMC3763457; doi:10.1038/cddis.2013.296)
Supplement: Supplementary Table S2 [file cddis2013296x4.doc]

**Table S2.** **Primers for quantitative real time PCR.**

| Target Gene |  | Sequence (5’🡪3’) | Amplicon size (nt) |
| --- | --- | --- | --- |
| **18S rRNA[1]** | Fwd: | TGAGAAACGGCTACCACATC | 112 |
|  | Rev: | TTACAGGGCCTCGAAAGAGT |  |
| **TNFSF12-13** | Fwd: | GCCAGATCGGGGAGTTTATAGT | 71 |
|  | Rev: | CAGGGCATCGGAACTCTG |  |
| **TNFSF13** | Fwd: | AAGGGTATCCCTGGCAGAGT | 129 |
|  | Rev: | GCAGGACAGAGTGCTGCTT |  |
| **USP47** | Fwd: | ACCAACTGGTCCCGAAAGA | 109 |
|  | Rev: | TCTTTATCTGTCAAATGCAGAAAGTT |  |

1. Giricz O, Lauer-Fields JL, Fields GB (2008) The normalization of gene expression data in melanoma: investigating the use of glyceraldehyde 3-phosphate dehydrogenase and 18s ribosomal RNA as internal reference genes for quantitative real-time PCR. Analytical Biochemistry 380: 137-139.

**Table S3.** **shRNA and siRNA sequences for TNFSF12-13 and TNFSF13.**

| **Oligo ID** | **shRNA^*^** | **siRNA** |
| --- | --- | --- |
| V2HS_17313 | GCCGCCCTCTGCTAGGGAA |  |
| V2HS_17314 | GATATTCTGAGTGTCATAA |  |
| V2HS_17316 | GGTGCCTTCGCAGTCAAAT |  |
| V2HS_17317 | GAGACTCTATTCCGATGTA |  |
| V2HS_17318 | CTCCAGAGATGTAGCTATT |  |
| J-032530-05 |  | GGGCAAGGGCGAAACUUAA |
| J-032530-06 |  | GCAGGUGUCUUCCAUUUAC |
| J-032530-07 |  | UGACAGAGGUGAUGUGGCA |
| J-032530-08 |  | GGAGUUUAUCUGCUGUAUA |
| J-011523-05 |  | GGGCAAGGGCGAAACUUAA |
| J-011523-06 |  | GCAGGUGUCUUCCAUUUAC |
| J-011523-07 |  | UGACAGAGGUGAUGUGGCA |
| J-011523-08 |  | GGAGUUUAUCUGCUGUAUA |

* shRNA targets both TNFSF12-13 and TNFSF13

**Table S4.** **shRNA and siRNA sequences for USP47.**

| **Oligo ID** | **shRNA** | **siRNA** |
| --- | --- | --- |
| V2HS_174637 | GAATCTGTCTTGAAACCAA |  |
| V2HS_174639 | CGCAATACATGCAAGATAA |  |
| V2HS_174641 | GGATTCCTTTGGATGATAT |  |
| V2HS_174642 | GATTTAGACTGGAATCCTA |  |
| V2HS_218228 | CAATGACTTGCTATTTGAA |  |
| J-006093-05 |  | GCAACGAUUUCUCCAAUGA |
| J-006093-06 |  | CAACAUGUCAGCAGGAUAA |
| J-006093-07 |  | GCUGUCGCCUUGUUAAAUA |
| J-006093-08 |  | CGCAAUACAUGCAAGAUAA |
