# Supplementary material for: Knockdown of specific host factors protects against influenza virus-induced cell death
Source: Cell Death Dis. 2013 Aug 15;4(8):e769–. doi: 10.1038/cddis.2013.296 (PMC3763457; doi:10.1038/cddis.2013.296)
Supplement: Supplementary Legend [file cddis2013296x5.doc]

**Supplementary Tables and Figure Legends**

**Table S1. Overlap between various influenza virus RNAi screens.**

**Table S2. Primers for quantitative real time PCR.**

**Table S3. shRNA and siRNA sequences for TNFSF12-13 and TNFSF13.**

**Table S4. shRNA and siRNA sequences for USP47.**

**Figure S1. Efficiency of protein knockdown in A549 cells. TNFSF12-13, TNFSF13, and USP47 transcripts were determined by real time PCR for TNFSF12-13 (A) stably transduced shRNA cells (P<0.001) or (B) siRNA-transfected cells (P<0.001); for TNFSF13 (C) stably transduced shRNA cells (P<0.001) or (D) siRNA-transfected cells (P<0.001); for USP47 (E) stably transduced shRNA cells (P<0.05) or (F) siRNA-transfected cells (P<0.001) and non-targeting (NSi) shRNA or siRNA control cells, respectively. C_t_ values were normalized to 18S rRNA control and compared to non-targeting control.** Shown are the means from duplicate runs with error bars representing standard deviation. USP47 protein knockdown was confirmed with Western blot in (G) shRNA- and (H) siRNA-treated cells.

**Figure S2. Viability of knockdown cells. Viability of knockdown cells was assessed by WST-1 for (A) stably transduced TNFSF12-13/TNFSF13 and (B) USP47 shRNA cells, (C) TNFSF12-13, (D) TNFSF13, and (E) USP47 siRNA-treated cells.** NSi is nontargeting shRNA or siRNA control. Error bars represent the mean + standard error of the mean from 3 independent replicates.
